# Soy Intake During Childhood and/or Adolescence and Adult Breast Cancer: An Examination of the Early Soy Intake Hypothesis

**DOI:** 10.3390/nu18111820

**Published:** 2026-06-04

**Authors:** Mark Messina, Alison M. Duncan

**Affiliations:** 1 Soy Nutrition Institute Global, P.O. Box 37, Jefferson City, MO 65102, USA; 2 Department of Human Health Sciences, University of Guelph, 50 Stone Road East, Guelph, ON N1G 2W1, Canada; amduncan@uoguelph.ca

**Keywords:** soy, isoflavones, genistein, breast cancer prevention, girls, early soy intake hypothesis

## Abstract

Breast cancer is one of the leading causes of cancer incidence and mortality in many countries worldwide although there is considerable geographic variation. Diet is thought to impact risk of developing breast cancer but identifying specific dietary factors involved in the etiology of this disease has proven difficult. The two primary factors that initially led to an interest in soy are the historically low breast cancer incidence and mortality rates in Japan and the uniquely high concentration of isoflavones in soybeans and foods derived from this legume. Isoflavones bind to both estrogen receptors although preferentially to estrogen receptor-β. Prospective cohort studies indicate that isoflavone intake is associated with a reduced risk of developing breast cancer, but randomized controlled trials in which the impact of soy and isoflavones on markers of breast cancer risk has been evaluated are not supportive of this protective association. It may be that for isoflavones to reduce risk, intake needs to occur during childhood and/or adolescence. The notion that consuming soy early in life reduces risk of adult breast cancer, herein referred to as the “early soy intake hypothesis” (ESIH), was proposed >30 years ago. The results of rodent studies and retrospective observational studies that examined incidence and/or markers of breast cancer risk support the ESIH. However, a lack of randomized controlled trials precludes a clear recommendation for soy consumption during childhood and/or adolescence specifically for breast cancer prevention. Although soy foods provide high-quality protein and a variety of nutrients and can be part of a healthy diet for young and adolescent girls, more research is needed to advance the ESIH.

## 1. Introduction

Approximately 1 in 8 US women will develop breast cancer in their lifetime and, in 2026, estimates are that over 300,000 women will be diagnosed with this disease, and more than 42,000 will die from it [1]. The average age of breast cancer diagnosis is 62. These sobering statistics explain the intense research effort conducted over the past several decades to develop improved treatment modalities [2] and to identify risk factors for developing breast cancer [3]. Diet is considered a lifestyle factor that can affect both the risk of developing breast cancer [4] and breast cancer prognosis [5].

Over the past three decades, soy foods and soy components (e.g., soy protein, isoflavones) have been rigorously investigated [6,7,8]. In addition to an abundance of observational studies, it is estimated that more than 600 randomized controlled trials (RCTs) have been conducted. Much of this research has focused on chronic disease prevention, incidence in the case of observational studies, and markers of risk, in the case of RCTs. But it is the relationship between soy and breast cancer, and more specifically, the impact of soybean isoflavone intake on risk of developing breast cancer, and breast cancer prognosis, that has attracted particular attention. In this perspective, evidence is reviewed relative to the hypothesis that consuming soy during childhood and/or adolescence reduces breast cancer risk later in life.

### 1.1. Isoflavones and Mammary Cancer

Isoflavones are naturally occurring compounds present in a wide range of foods, but in uniquely rich amounts in soybeans and foods derived from this legume, which accounts for why average isoflavone intake in Japan among individuals consuming a traditional diet is approximately 30–50 mg/d [9], but fewer than 3 mg/d in Europe [10] and the United States [11]. The three soybean isoflavone aglycones, genistein, daidzein and glycitein, and their respective glycosides (the predominant form in unfermented soy), account for about 50, 40, and 10%, respectively, of total isoflavone content [12].

It is fair to say that modern research interest in soy was stimulated by animal work showing that soybean isoflavones inhibited the development of mammary cancer in rats [13], as this finding, along with the low historical breast cancer incidence [14] and mortality [14] rates in soy food-consuming countries, led the US National Cancer Institute in the early 1990s to fund isoflavone research [15]. But less than a decade later, research led to concerns that isoflavone intake might worsen the prognosis of women with breast cancer. The primary soybean isoflavone genistein was found to stimulate the growth of existing estrogen-sensitive mammary tumors in ovariectomized athymic mice [16]. Isoflavone research published over the next 15 years involving this basic rodent model continued to raise concern [17,18,19].

Collectively, this research demonstrated that in ovariectomized athymic mice, genistein functions as estrogen receptor (ER) agonist, thereby stimulating the growth of the estrogen-sensitive tumors. For that reason, genistein inhibited the efficacy of tamoxifen [20] and the aromatase inhibitor, letrozole [21]. In contrast, genistein was without effect in this model when mice were implanted with ER- breast cancer cells [22]. Also, it is notable that in a similar mouse model, genistein was without effect on the growth of existing estrogen-sensitive tumors [23]. The authors attributed this lack of effect to the MCF-7 cells that were implanted into the mice in their study being less estrogen sensitive, as a result of the cells being cultured in medium absent of estrogen prior to implantation, than the highly sensitive MCF-7 cells implanted into the mice in which tumor stimulation was observed [24,25].

These two seemingly opposing outcomes observed in rodents—inhibiting development of mammary tumors and stimulating the growth of existing tumors—are not necessarily in conflict mechanistically as the timing of isoflavone exposure (pre vs. post tumor occurrence) could determine the effect on carcinogenesis. A potentially relevant example of the importance of timing in relation to breast cancer risk is age at first full-term pregnancy, as pregnancy earlier in life is thought to decrease long-term breast cancer risk, whereas pregnancy later in life is thought to increase it [26,27].

### 1.2. Impact of Adult Soy Food Intake on Breast Cancer Risk and Prognosis

In contrast to the rodent research raising concerns [16,17,18,19,24,25], beginning in 2009 [28], cohort studies began to show that postdiagnosis soy intake improves breast cancer prognosis [29,30,31]. Accordingly, in 2022, the American Cancer Society concluded that “… postdiagnosis soy isoflavone intake is associated with a lower risk of recurrence” [32] and in 2023, the American Institute for Cancer Research/World Cancer Research Fund International identified soy food intake as one of five factors, the others being healthy body mass index (BMI), healthy eating patterns, physical activity and dietary fiber intake, that may reduce the mortality of breast cancer patients [33]. In addition, the authors of a recently published comprehensive review concluded that regardless of their treatment status (i.e., tamoxifen and/or aromatase inhibitors), evidence indicates that women with breast cancer can safely consume soy foods [31].

Cohort studies also indicate that isoflavone intake is associated with a lower risk of developing breast cancer. In a recent dose–response meta-analysis of seven cohort studies, Yang et al. [34] found that, depending upon the statistical model applied, each 10 mg/d increase in isoflavone intake was associated with a statistically significant 3.2% or 6.8% decrease in breast cancer risk. Thus, observational research supports the results of rodent studies that show mammary cancer is inhibited by isoflavones [13] but refutes results raising concern [16,17,18,19,24,25]. However, clinical research examining markers of breast cancer risk is unsupportive of benefit in that isoflavones provided in the form of supplements, soy protein or soy foods do not affect mammographic density (n = 14 studies) or breast cell proliferation (n = 6 studies) (see reference for review) [31].

The results of these clinical studies, which were conducted in healthy women and women at increased risk of or living with breast cancer, are a strong argument for safety, and like the observational research, counter concerns based on animal studies. But they also strongly argue against a protective effect, because in contrast to the lack of effect of isoflavones, tamoxifen and aromatase inhibitors reduce breast tissue density [35] and breast cell proliferation [36,37].

### 1.3. Proposed Mechanisms for the Protective Effect of Adult Soy Intake Against Breast Cancer

Over the years, two primary mechanisms, one estrogen-dependent and the other not, have been proposed to explain the hypothesized breast cancer-protective effects of isoflavones. One, which has largely fallen out of favor, is the inhibitory effect of genistein on the activity of tyrosine protein kinases, a family of enzymes overexpressed in cancer cells [38,39]. Clinical support for this mechanism is lacking as this inhibition has not been demonstrated in humans, which is consistent with the circulating concentrations of genistein in response to soy food consumption, relative to the much higher in vitro concentrations required for this action.

The other mechanism involves an interaction between isoflavones and ERs. Theoretically, isoflavones may exert anti-proliferative effects through differential modulation of ER subtypes—by competing with endogenous estrogens for binding to ERα, thereby attenuating ERα-mediated proliferative signaling [40], and/or by preferentially binding to ERβ [41], activation of which is generally associated with anti-proliferative pathways [40].

However, in clinical studies, rarely, if ever, have isoflavones been shown to inhibit the action of estrogen. In fact, a recently published systematic review and meta-analysis of RCTs challenges even the notion that isoflavones exert estrogen-like effects [42], despite many of the proposed benefits of these soybean constituents for postmenopausal women (e.g., menopause symptom relief, decreased bone resorption/increased bone formation, improved memory) which have varying degrees of support—being assumed to result from the estrogen-like properties of isoflavones [7].

### 1.4. The Early Soy Intake Hypothesis (ESIH)

Is there still a reasonable basis for speculating that soy/isoflavones reduce breast cancer risk since as noted above, the clinical research [31] fails to show favorable effects on markers of breast cancer risk? There is, when one recalls the hypothesis proposed in 1995, that isoflavone intake, specifically when occurring early in life, is protective against this disease [43,44]. This hypothesis is consistent with the school of thought, as was highlighted by Colditz and Frazier [45] in that same year, that early life events profoundly impact later risk of developing breast cancer.

Subsequently published research by Peng et al. [46] corroborated the rodent research upon which the hypothesis was based in that early exposure to genistein inhibited chemically induced mammary cancer. The ESIH, which has been discussed over the years [47,48], has epidemiologic support from the four studies—two from China [49,50] and two from the US [51,52]—that have evaluated this hypothesis. However, the most recent of these studies was published a decade ago [50]. Note that both US studies involved women of Asian ethnicities as it has been argued that soy intake among the US population is too low to exert a physiological effect [53].

The limited relevant observational research likely stems from the difficulty of generating data to test the ESIH. Short of conducting a many-decade-long prospective cohort study, testing the ESIH requires a retrospective design. For this reason, the results of a recently published retrospective study by Ho and colleagues [54], from the Chinese University of Hong Kong, are so important.

Ho et al. [54] did not examine soy intake and breast cancer risk, but rather mammographic density. Mammographic density represents the amount of fibrous and glandular tissue compared to fatty tissue in the breast and is a well-established marker of breast cancer risk [55,56,57]. Recently published research continues to support mammographic density as a surrogate breast cancer marker. For example, in a nested case-control cohort study sampled from the Joanne Knight Breast Health Cohort, Jiang et al. [58] found that the rate of change in breast density was associated with the risk of subsequent breast cancer. Also, Mulder et al. [59] found that integrating mammographic density with established risk factors and polygenic risk score may enhance breast cancer risk stratification among European-ancestry women, supporting its potential for clinical utility.

For the analysis by Ho et al. [54], 815 premenopausal women with a mean age of 40.9 were interviewed to determine their soy intake during the past 12 months (current), and during the time periods of 20–34, 13–18 and 6–12 years of age. Mean soy protein (g/d) and isoflavone intake (mg/d) for these four time periods was 10.3 and 22.1, 12.6 and 27.2, 11.3 and 25.1, and 9.3 and 21.4, respectively. The similarity in soy intake between middle-aged women and young girls, despite differences in caloric intake is striking, and may reflect Westernization of the Hong Kongese diet. Soy food intake was assessed using a validated food frequency questionnaire that included 32 soy foods. It is notable that to assess the validity of participants’ recall of soy food intake in early life, separate interviews were conducted for participants’ food preparers (N = 76) [mothers (89%), sisters (11%)].

When comparing high vs. low soy protein intake, and high versus low isoflavone intake during these four periods of life, mammographic density was statistically significantly lower for the periods 6–12 and 13–18 years of age, but not for the other two. More specifically, the percentage differences in mammographic density for high vs. low soy protein intake for the four time periods (current, 20–34, 13–18 and 6–12 years of age) were −1.47, −2.69, −5.84, and −6.35, respectively, and for high vs. low isoflavone intake, they were −1.28, −2.53, −5.91 and −6.50, respectively.

There appears to be a stepwise increase in the mammographic density percentage difference, indicating greater benefit, between the high and low soy protein and isoflavone intakes with decreasing age. However, Ho et al. [54] did not statistically analyze this trend. Importantly, as was pointed out by Ho et al. [54], the difference in mammographic density due to soy intake is likely clinically relevant because reductions of a similar magnitude have been noted in association with adolescent stature [60], the menopausal transition [61], conventional hormone therapy [62] and tamoxifen use [63].

Interestingly, in a small US observational study by Korde et al. [52], soy intake during 5–11 years of age was associated with a larger decrease in breast cancer risk than soy intake during 12–19 years of age (60 vs. 20%). However, the authors did not compare high soy intake throughout life with high soy intake only when young. It is noteworthy that in the study by Ho et al. [54], the mean difference in mammographic density when comparing high versus low lifetime soy protein intake (all four time periods) (−2.29%) and high versus low lifetime soy protein intake minus current intake (−6.20%) did not result in a greater decrease than high versus low soy protein intake during 6–18 years of age (−6.11%). Thus, in this study, later life soy intake does not appear to enhance the protective effects of early intake.

Nevertheless, the question of whether high lifetime soy intake reduces breast cancer risk to a greater extent than high soy intake only early in life remains an open one. In one of the previously cited US retrospective studies, the relative risks (RRs) for breast cancer associated with high soy intake during adolescence but not adulthood, and high soy intake during both time periods, were 0.72 and 0.62, respectively [51].

The lack of significant association between current soy protein/isoflavone intake and mammographic density in the study by Ho et al. [54] is consistent with the lack of significant effect observed in clinical studies noted previously [31]. The lower mammographic density associated with early intake is consistent with work by Maskarinec et al. [64], who used multilevel modeling to show that soy intake during childhood and adolescence was related to lower percent mammographic densities whereas soy consumption during adulthood predicted significantly higher densities in the first mammogram studied (mean age, 57 years). The latter finding disagrees with the study by Ho et al. [54] and the clinical research cited previously [31].

A potentially important observation is that epidemiologic research indicates that early-age pregnancy specifically protects against ER+ and progesterone receptor (PR)+ breast cancers with no effects on the risk of ER− and PR− breast cancers [65]. However, little information is available about early soy intake and risk of breast cancer subtype. Of the four observational studies that evaluated early soy intake and breast cancer risk, two sub-analyzed the results according to menopausal status. In the study by Shu et al. [49], when comparing quintile 5 adolescent soy intake with quintile 1, RRs for pre- and postmenopausal breast cancer were very similar. In contrast, when comparing high versus low juvenile soy intake, Baglia et al. [50] found the RR was much lower for pre- compared to postmenopausal breast cancer. In the studies by Korde et al. [52] and Wu et al. [51,66], 74% and 45% of the breast cancer cases were premenopausal, respectively, but the data were not sub-analyzed according to menopausal status. Finally, the recent research by Ho et al. [54] involved premenopausal women.

Before concluding this section, it warrants emphasis that the observational studies supporting the ESIH, not surprisingly, utilized a retrospective design. Not only were individuals asked to recall dietary intake many years earlier, but during a very young period of life. In some cases when available, the mothers of the women in these studies also provided intake data for their daughter. Thus, the self-reporting of dietary intake is subject to error and, potentially, recall bias. Also, it is possible that the composition of the foods consumed many decades earlier may be different from the current versions which were used to analyze the isoflavone content of the diet. Nevertheless, retrospective assessment of dietary intake can be a reliable indicator of past intake, and retrospective studies assessing dietary intake are routinely used to gain insight about the impact of diet on health [67].

### 1.5. Early Soy Intake Hypothesis: Possible Mechanisms

Several intriguing but highly speculative mechanisms may explain the hypothesized protective effect of early soy intake against breast cancer. Research suggests that exposure to endogenous hormones and environmental chemicals during early development affects breast cancer incidence [68,69,70]. Warri et al. [71] proposed that the effects on mammary gland morphology and signaling pathways induced by pubertal exposure to genistein mimic those induced by the estrogenic environment of early first pregnancy, although Muenst et al. [72] attributed the protective effects of pregnancy at early age to production of progesterone-responsive cells and epithelial Wnt signaling.

As noted previously, when activated, ERα and ERβ typically result in proliferative and anti-proliferative effects, respectively [73]. Thus, findings that isoflavones increase expression of the latter [74] and decrease expression of the former [75], suggests isoflavones would produce an anti-proliferative effect, but how this change would affect the likelihood of girls developing breast cancer later in life is unclear. Isoflavones have also been shown to upregulate BRCA1 expression [76], which could offer protection against breast cancer as this tumor suppressor gene is involved in the repair of DNA damage [77].

There is much interest in understanding how epigenetic alterations influence breast cancer risk [78]. Noteworthy is that in two preclinical patient-derived xenograft orthotopic mouse models of triple negative breast cancer, genistein modified expression levels of key epigenetic-associated genes [79]. This work aligns with that by Guo et al. [80], who found long-term prediagnosis soy intake was associated with increased expression of tumor suppressors and decreased expression of oncogenes, especially cell growth-related genes, in breast tumors. Also, Jadhav et al. [81] found that prepubertal exposure to genistein hypomethylated two genes in the mammary glands of rats associated with improved long-term survival of breast cancer patients. Thus, there is evidence from preclinical research that isoflavones may cause epigenetic changes. However, in a pilot study, after a 5-day soymilk treatment, no major general epigenetic reprogramming could be found in glandular tissue DNA methylome in women undergoing an aesthetic breast reduction [82]. Given the short exposure, and that this pilot study involved adults, its relevance to the ESIH is unclear.

### 1.6. Impact of Soy Intake on Puberty Onset and Thyroid Function

The ESIH is particularly compelling from a public health standpoint, as the proposed benefit is associated with a modest and achievable intake—approximately one to two daily servings of a traditional Asian soy food. One serving, such as 1 cup soymilk, 3–4 ounces tofu or edamame, provides approximately 20 to 25 mg isoflavones. However, al-though the ESIH maintains that isoflavone intake when young lowers breast cancer risk, isoflavones are not without controversy. Most relevant to the ESIH is the concern that isoflavone intake by young girls may advance puberty onset [83]. There is a worldwide trend toward earlier puberty in girls, although this trend is occurring in countries where soy foods are, as well as countries where soy foods are not, part of the traditional diet [84]. The evidence discussed in this manuscript supporting the ESIH indirectly argues against soy consumption advancing the age of menarche onset, since earlier menarche is associated with an increased risk of breast cancer [85].

Interestingly, epidemiologic studies have found that soy intake is associated with both earlier [86,87,88] and later [89,90] age of puberty onset whereas a large US cross-sectional study found no association between soy intake and age of menarche onset among Seventh-day Adventist girls [91]. A comprehensive review published in 2022 concluded that observational studies have not shown a consistent association between soy isoflavone intake and early development in girls or boys [92]. Finally, of relevance to puberty onset is a recently published analysis of the Legacy Girls Study, which revealed that high urinary glucocorticoid metabolites, high BMI and stress were associated with earlier thelarche, a marker of puberty [93].

There has also been concern, based primarily on animal studies, that soy food intake may adversely affect thyroid function [94]. However, in 2015, the European Food Safety Authority [95] and, in 2018, the Permanent Senate Commission on Food Safety of the German Research Foundation [96], concluded isoflavones do not affect thyroid function in postmenopausal women (the only population group studied). In alignment, a meta-analysis of RCTs involving adults, published in 2019, found that neither soy nor isoflavone intake affected circulating levels of thyroxine (T4) or triiodothyronine (T3) [97]. Perhaps more relevant to the ESIH is a small 8-week trial by Zung et al. [98] involving hypercholesterolemic children aged 5 to 11 years, which found that neither a low (16 mg/d) nor high (48 mg/d) dose of isoflavones affected levels of free T4 or T3 or thyroid stimulating hormone.

With respect to hypothyroid patients, soy protein likely inhibits the absorption of levothyroxine, but this is true for food in general and many dietary supplements, herbs and drugs [99]. Hypothyroid patients do not have to avoid all soy if there is a sufficient time interval between soy consumption and levothyroxine ingestion. Recommendations are to consume the medication ~1 h before breakfast or to wait as long as fours after eating [100]. Alternatively, as long as soy intake occurs in a consistent manner, the dose of levothyroxine can be adjusted appropriately so if necessary [101].

## 2. Summary and Conclusions

In closing, the evidence from Ho et al. [54] and multiple preclinical [43,44,46] and observational studies [49,50,51,52] examined in this commentary are supportive of the ESIH. However, because RCTs are lacking, it is premature to recommend that young and adolescent girls consume soy foods solely for the purpose of reducing breast cancer risk. On the other hand, soy foods provide high-quality protein [102], a variety of vitamins and minerals [84] and are typically low in saturated fat, providing ample reasons for young and adolescent girls to consume soy. Whether the effect of early soy consumption on breast cancer risk can be added to that list remains to be determined. Clearly though, the ESIH warrants further study; in addition to more retrospective studies, this research could include longitudinal or intervention studies assessing gene expression and other relevant biomarkers in young and adolescent girls consuming soy [103]. Given the global public health burden breast cancer represents and the relative lack of clear evidence for dietary factors that affect breast cancer risk, this research can be considered a high priority.

## Data Availability

No new data were created or analyzed in this study.

## Abbreviations

BMI, body mass index; BRCA1, breast cancer gene 1; ESIH, early soy intake hypothesis; ER+, estrogen receptor-positive; ER−, estrogen receptor-negative; PR−, progesterone receptor-negative; PR+, progesterone receptor positive; RCTs, randomized controlled trials; RR, relative risk.
